# 1,3-Phenyl­enediammonium dinitrate

**DOI:** 10.1107/S1600536809039166

**Published:** 2009-10-03

**Authors:** Bobby Portis, Kalpana R. Dey, Musabbir A. Saeed, Douglas R. Powell, Md. Alamgir Hossain

**Affiliations:** a Department of Chemistry and Biochemistry, 1400 J. R. Lynch St, PO Box 17910, Jackson State University, Jackson, MS 39217-0510, USA; b Department of Chemistry and Biochemistry, University of Oklahoma, 620 Parrington Oval, Room 208, Norman, OK 73019-3051, USA

## Abstract

In the title compound, C_6_H_10_N_2_^2+^·2NO_3_^−^, the dication lies on a crystallographic twofold rotation axis. The nitrate ions are linked to the dications though N—H⋯O hydrogen bonds, forming a three-dimensional network.

## Related literature

For general background to polyamines, see: Bianchi *et al.* (1997 ▶); Ilioudis *et al.* (2002 ▶); Hossain (2008 ▶). For related structures, see: Anderson *et al.* (2006 ▶; Gawlicka-Chruszcz & Stadnicka (2002 ▶); Soumhi & Jouini (1995 ▶); Wang *et al.* (2007 ▶).
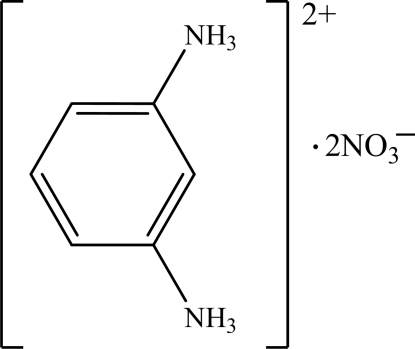


## Experimental

### 

#### Crystal data

C_6_H_10_N_2_^2+^·2NO_3_^−^
*M* *_r_* = 234.18
Monoclinic, 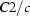

*a* = 16.2548 (12) Å
*b* = 9.6212 (8) Å
*c* = 7.1070 (6) Å
β = 115.506 (6)°
*V* = 1003.14 (14) Å^3^
*Z* = 4
Cu *K*α radiation
μ = 1.22 mm^−1^
*T* = 100 K
0.53 × 0.50 × 0.24 mm

#### Data collection

Bruker APEX CCD area-detector diffractometer
Absorption correction: multi-scan (*SADABS*; Sheldrick, 2007 ▶) *T*_min_ = 0.562, *T*_max_ = 0.761
5278 measured reflections
942 independent reflections
882 reflections with *I* > 2σ(*I*)
*R*_int_ = 0.036

#### Refinement

*R*[*F* ^2^ > 2σ(*F* ^2^)] = 0.033
*wR*(*F* ^2^) = 0.092
*S* = 1.01
942 reflections
84 parameters
H atoms treated by a mixture of independent and constrained refinement
Δρ_max_ = 0.26 e Å^−3^
Δρ_min_ = −0.20 e Å^−3^

### 

Data collection: *SMART* (Bruker, 1998 ▶); cell refinement: *SAINT* (Bruker, 1998 ▶); data reduction: *SAINT*; program(s) used to solve structure: *SHELXTL* (Sheldrick, 2008 ▶); program(s) used to refine structure: *SHELXTL*; molecular graphics: *SHELXTL*; software used to prepare material for publication: *SHELXTL*.

## Supplementary Material

Crystal structure: contains datablocks global, I. DOI: 10.1107/S1600536809039166/ci2920sup1.cif

Structure factors: contains datablocks I. DOI: 10.1107/S1600536809039166/ci2920Isup2.hkl

Additional supplementary materials:  crystallographic information; 3D view; checkCIF report

## Figures and Tables

**Table 1:** Hydrogen-bond geometry (Å, °)

*D*—H⋯*A*	*D*—H	H⋯*A*	*D*⋯*A*	*D*—H⋯*A*
N5—H5*A*⋯O1*A*	0.94 (2)	1.87 (2)	2.7955 (15)	168 (2)
N5—H5*B*⋯O1*A*^i^	0.92 (2)	1.95 (2)	2.8416 (16)	163 (2)
N5—H5*C*⋯O3*A*^ii^	0.92 (2)	1.96 (2)	2.8626 (16)	167 (2)

Symmetry codes: (i) 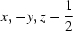; (ii) 
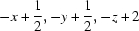
.

**Fig. 1.**
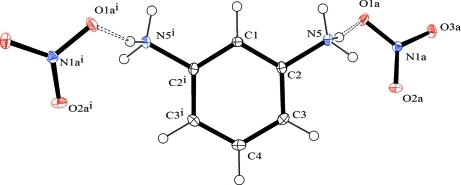
The formula unit of the title compound, showing the atom-numbering scheme. Displacement ellipsoids are drawn at the 50% probability level. Dashed lines indicate hydrogen bonding interactions. Symmetry code: (i) -x, y, 1/2 -z.

**Fig. 2.**
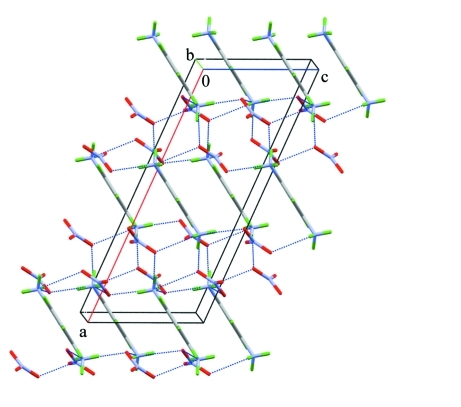
Crystal packing of the title compound, viewed along the b axis.

## supplementary crystallographic information

### Comment

Simple polyammonium ions are known as excellent hydrogen bond donors for a variety of anions in particular for oxoanions, forming supramolecular aggregates with hydrogen bonding networks (Ilioudis *et al.*, 2002). Indeed, a difunctional or trifunctional polyamine is widely used as an essential building block for a macrocyclic based host, and acts as major binding components for a negatively charged anion (Bianchi *et al.*, 1997; Hossain, 2008). In this study, we used a simple 1,3-phenylenediamine to prepare an adduct with nitric acid. We report, herein, the crystal structure of the title compound in which the nitrate anions are connected to the cationic units through hydrogen bonding interactions.

X-ray analysis of the nitrate salt reveals that both amino groups are protonated to form a dication and crystallized with two nitrate anions. In the crystal lattice, each diaction is surrounded by two symmetry related nitrate anions (Fig. 1). Each amino group is engaged in coordinating nitrate anions through N—H ···O bonds ranging from 2.7955 (15) to 2.8626 (16) Å (see Table 1). The crystal structure viewed along the *b* axis shows that the cations are arranged antiparallel to one another along the *c* axis in which two adjacent aromatic units are separated at 7.024 Å (Fig. 2). Therefore, there is no π-π stacking involved. The nitrates serve as linkers of the two adjacent aromatic units by hydrogen bonding networks along the *b* axis.

### Experimental

To a solution of 1,3-phenylenediamine (0.1 g) in CH_3_OH (2 ml) was added a few drop of nitric acid. The white precipitate formed immediately was filtered and washed with diethyl ether. Yield: 80%. M.P. 150.5°C. ^1^H NMR (500 MHz, D_2_O, TSP): *δ* 7.15 (m, *J *= 4 Hz,1*H*, Ar*H*), 6.68 (d, *J *= 8 Hz, *J *= 2 Hz, 2H, Ar*H*), 6.62 (t, *J*= 2 Hz, 1H, Ar*H*). Crystals suitable for X-ray crystallography were obtained by recystallization from a methanolic solution of the salt and isolated after seven days keeping the solution under Et_2_O diffusion in a desiccator.

### Refinement

H atoms bonded to carbons were positioned geometrically and refined using a riding model, with C-H = 0.99 Å and *U*_iso_(H) = 1.2 *U*_eq_(C). H atoms bonded to N atoms were located in a difference map and their positional parameters were refined, with *U*_iso_(H) = 1.2 *U*_eq_(N).

### Crystal data

**Table:**

C_6_H_10_N_2_^2+^·2NO_3_^−^	*F*(000) = 488
*M**_r_* = 234.18	*D*_x_ = 1.551 Mg m^−^^3^
Monoclinic, *C*2/*c*	Cu *K*α radiation, λ = 1.54178 Å
Hall symbol: -C 2yc	Cell parameters from 3468 reflections
*a* = 16.2548 (12) Å	θ = 5.5–69.5°
*b* = 9.6212 (8) Å	µ = 1.22 mm^−^^1^
*c* = 7.1070 (6) Å	*T* = 100 K
β = 115.506 (6)°	Block, colorless
*V* = 1003.14 (14) Å^3^	0.53 × 0.50 × 0.24 mm
*Z* = 4	

### Data collection

**Table:**

Bruker APEX CCD area-detector diffractometer	942 independent reflections
Radiation source: fine-focus sealed tube	882 reflections with *I* > 2σ(*I*)
graphite	*R*_int_ = 0.036
φ and ω scans	θ_max_ = 69.5°, θ_min_ = 5.5°
Absorption correction: multi-scan (*SADABS*; Sheldrick, 2007)	*h* = −19→18
*T*_min_ = 0.562, *T*_max_ = 0.761	*k* = −11→11
5278 measured reflections	*l* = −8→8

### Refinement

**Table:**

Refinement on *F*^2^	Secondary atom site location: difference Fourier map
Least-squares matrix: full	Hydrogen site location: inferred from neighbouring sites
*R*[*F*^2^ > 2σ(*F*^2^)] = 0.033	H atoms treated by a mixture of independent and constrained refinement
*wR*(*F*^2^) = 0.092	*w* = 1/[σ^2^(*F*_o_^2^) + (0.054*P*)^2^ + 1.07*P*] where *P* = (*F*_o_^2^ + 2*F*_c_^2^)/3
*S* = 1.00	(Δ/σ)_max_ = 0.001
942 reflections	Δρ_max_ = 0.26 e Å^−^^3^
84 parameters	Δρ_min_ = −0.20 e Å^−^^3^
0 restraints	Extinction correction: *SHELXTL* (Sheldrick, 2008), Fc^*^=kFc[1+0.001xFc^2^λ^3^/sin(2θ)]^-1/4^
Primary atom site location: structure-invariant direct methods	Extinction coefficient: 0.0046 (5)

### Fractional atomic coordinates and isotropic or equivalent isotropic displacement parameters (Å^2^)

**Table:**

	*x*	*y*	*z*	*U*_iso_*/*U*_eq_	
N1A	0.16571 (7)	0.19642 (12)	1.08086 (17)	0.0129 (3)	
O1A	0.11343 (7)	0.11334 (11)	0.94063 (14)	0.0171 (3)	
O2A	0.17425 (7)	0.31761 (10)	1.03487 (16)	0.0205 (3)	
O3A	0.20794 (7)	0.15171 (11)	1.26369 (14)	0.0170 (3)	
C1	0.0000	0.16909 (19)	0.2500	0.0119 (4)	
H1	0.0000	0.0703	0.2500	0.014*	
C2	0.06684 (9)	0.24329 (14)	0.40956 (19)	0.0125 (3)	
C3	0.06855 (9)	0.38745 (15)	0.4119 (2)	0.0148 (3)	
H3	0.1156	0.4364	0.5218	0.018*	
C4	0.0000	0.4588 (2)	0.2500	0.0167 (4)	
H4	0.0000	0.5575	0.2500	0.020*	
N5	0.13746 (8)	0.16679 (12)	0.58086 (17)	0.0136 (3)	
H5A	0.1261 (12)	0.1620 (17)	0.700 (3)	0.016*	
H5B	0.1409 (11)	0.079 (2)	0.535 (3)	0.016*	
H5C	0.1918 (13)	0.2147 (19)	0.624 (3)	0.016*	

### Atomic displacement parameters (Å^2^)

**Table:**

	*U*^11^	*U*^22^	*U*^33^	*U*^12^	*U*^13^	*U*^23^
N1A	0.0125 (6)	0.0140 (6)	0.0121 (6)	0.0001 (4)	0.0053 (4)	−0.0013 (4)
O1A	0.0187 (5)	0.0179 (5)	0.0115 (5)	−0.0058 (4)	0.0034 (4)	−0.0038 (4)
O2A	0.0234 (6)	0.0108 (5)	0.0243 (6)	−0.0001 (4)	0.0075 (4)	0.0016 (4)
O3A	0.0154 (5)	0.0232 (6)	0.0100 (5)	−0.0005 (4)	0.0032 (4)	0.0017 (4)
C1	0.0139 (9)	0.0107 (9)	0.0117 (9)	0.000	0.0062 (7)	0.000
C2	0.0122 (7)	0.0161 (7)	0.0097 (6)	0.0005 (5)	0.0053 (5)	0.0011 (5)
C3	0.0157 (7)	0.0151 (7)	0.0138 (7)	−0.0031 (5)	0.0065 (6)	−0.0034 (5)
C4	0.0215 (10)	0.0119 (9)	0.0191 (9)	0.000	0.0112 (8)	0.000
N5	0.0133 (6)	0.0149 (6)	0.0098 (6)	−0.0005 (4)	0.0022 (5)	−0.0005 (4)

### Geometric parameters (Å, °)

**Table:**

N1A—O2A	1.2348 (16)	C3—C4	1.3901 (16)
N1A—O3A	1.2556 (15)	C3—H3	0.95
N1A—O1A	1.2747 (15)	C4—H4	0.95
C1—C2	1.3838 (16)	N5—H5A	0.943 (19)
C1—H1	0.95	N5—H5B	0.92 (2)
C2—C3	1.387 (2)	N5—H5C	0.924 (19)
C2—N5	1.4621 (16)		
			
O2A—N1A—O3A	121.59 (11)	C4—C3—H3	120.7
O2A—N1A—O1A	119.88 (11)	C3^i^—C4—C3	120.83 (18)
O3A—N1A—O1A	118.53 (11)	C3—C4—H4	119.6
C2^i^—C1—C2	117.89 (17)	C2—N5—H5A	112.7 (10)
C2—C1—H1	121.1	C2—N5—H5B	108.4 (11)
C1—C2—C3	122.02 (12)	H5A—N5—H5B	109.8 (14)
C1—C2—N5	118.72 (13)	C2—N5—H5C	108.7 (11)
C3—C2—N5	119.26 (11)	H5A—N5—H5C	104.8 (15)
C2—C3—C4	118.62 (12)	H5B—N5—H5C	112.5 (15)
C2—C3—H3	120.7		
			
C2^i^—C1—C2—C3	−0.49 (9)	N5—C2—C3—C4	−178.80 (10)
C2^i^—C1—C2—N5	179.28 (13)	C2—C3—C4—C3^i^	−0.47 (8)
C1—C2—C3—C4	0.97 (17)		

Symmetry codes: (i) −*x*, *y*, −*z*+1/2.

### Hydrogen-bond geometry (Å, °)

**Table:**

*D*—H···*A*	*D*—H	H···*A*	*D*···*A*	*D*—H···*A*
N5—H5A···O1A	0.94 (2)	1.87 (2)	2.7955 (15)	168 (2)
N5—H5B···O1A^ii^	0.92 (2)	1.95 (2)	2.8416 (16)	163 (2)
N5—H5C···O3A^iii^	0.92 (2)	1.96 (2)	2.8626 (16)	167 (2)

Symmetry codes: (ii) *x*, −*y*, *z*−1/2; (iii) −*x*+1/2, −*y*+1/2, −*z*+2.
